# Partial Codes Risk Whole Confusion: Characteristics and Outcomes of Pediatric Partial Code Orders

**DOI:** 10.3390/children13010106

**Published:** 2026-01-11

**Authors:** Rachel Jalfon, Brittany Cowfer, Shankari Kalyanasundaram, Deena R. Levine, Griffin Collins, Erica C. Kaye, Liza-Marie Johnson, R. Ray Morrison, Ashish Pagare, Meaghann S. Weaver

**Affiliations:** 1 Division of Palliative Care, Department of Oncology, St. Jude Children’s Research Hospital, Memphis, TN 38105, USAbrittany.cowfer@stjude.org (B.C.); deena.levine@stjude.org (D.R.L.); griffin.collins@stjude.org (G.C.); erica.kaye@stjude.org (E.C.K.); 2 Department of Palliative Care, University of Tennessee Health Science Center, Memphis, TN 38163, USA; 3 Analytic Services, Information Services, St Jude Children’s Research Hospital, Memphis, TN 38105, USA; shankari.kalyanasundaram@stjude.org (S.K.);; 4 Bioethics Program, St. Jude Children’s Research Hospital Memphis, TN 38105, USA; liza.johnson@stjude.org; 5 Division of Critical Care Medicine, Department of Pediatric Medicine, St. Jude Children’s Research Hospital, Memphis, TN 38105, USA; ray.morrison@stjude.org

**Keywords:** code status, pediatrics, ethics, end-of-life

## Abstract

**Objective**—Partial do-not-resuscitate (DNR) orders, directives specifying limited resuscitative efforts, are intended to align medical interventions with patient preferences. However, their complexity may introduce ambiguity, inconsistent care, and ethical challenges. **Design**—A retrospective review was conducted of inpatient partial code order entries over a three-year period at a single institution with a pediatric oncology and immunology cohort. Partial DNR orders were identified and categorized based on included or excluded interventions (chest compressions, defibrillation, intubation, mechanical ventilation, medications). Data was analyzed to assess the frequency, variation, and internal consistency of documented preferences as well as alignment with institutional definitions and clinical feasibility. **Results**—Partial DNR orders represented a small (n = 15, 7%) but notable proportion of total code status entries. Wide variability was observed in the combinations of permitted and withheld interventions, with orders containing internally conflicting instructions. Documentation of inconsistencies and unclear terminology were common, raising concerns about interpretability during emergent situations. **Conclusions**—Partial DNR orders demonstrate heterogeneity and potential for miscommunication. These findings suggest that while partial codes may reflect nuanced patient preferences, they pose operational and ethical risks that could compromise care clarity. Clinical implications are reviewed. These findings will guide institutional deliberations regarding whether to refine, restrict, or eliminate partial code order options to enhance patient safety and decision-making transparency.

## 1. Introduction

In some pediatric hospitals, code status order sets include the possibility for partial codes. Partial code refers to a limited DNR order whereby allowance is made for some components of resuscitation (such as chest compressions without shocking or without intubation) rather than a comprehensively packaged resuscitation effort [1]. A critique of partial codes centers on the lack of evidence to support their effectiveness [2]. In pediatric emergency centers, partial codes may create complications by de-standardizing resuscitation practices and risk undermining the integrity of protocols developed from evidence of full CPR versus DNR [3]. Partial codes introduce complexity into urgent, high-stakes settings. When a code is called and team members lack clarity on which resuscitation elements are authorized, the risk of error, delay, misinterpretation, and even chaos increases [4].

Large observation studies consistently show that conventional CPR has better neurologically favorable outcomes than compression-only CPR in children in out of hospital events, reflecting the predominantly respiratory etiology of pediatric arrests [5]. In-hospital survival rates from pediatric cardiac arrest shows survival to discharge rates of approximately 44%, while out-of-hospital cardiac arrest survival ranges from 6.6% in infants to 17.3% in adolescents [6]. If algorithm-based resuscitation events are often not successful for survival and neurologic well-being, then it is reasonable to expect partial codes to be even less beneficial.

Ethical concerns with partial codes also warrant consideration. On the surface, partial codes may appear to honor patient or parental (for age of minority) preferences, yet this places the onus of physiologic understanding on families who may lack the pre-requisite knowledge to make a truly informed decision [7]. If a partial code is essentially a symbolic gesture rather than a meaningful pathway to resuming spontaneous circulation based on the evidence-based algorithm of Pediatric Advance Life Support (PALS), it may subject patients to interventions that are harmful with little chance of benefit (e.g., chest compressions without intubation or defibrillation) [8]. At the system level, permitting variable code practices may challenge the consistency, trustworthiness, and equity of care. Patients with similar clinical states might receive different resuscitation approaches depending on how code status is framed.

To address these concerns, this project aimed to quantify the prevalence of partial DNR orders and characterize the preferences documented in a partial code order at a large free-standing pediatric hospital. This work focused on a pediatric subspeciality population (cancers, blood disorders, immunologic conditions) at a single institution. While the extent of including partial codes in electronic health record (EHR) systems is not known in a quantifiable way across other health systems, the project team is aware from their prior work in pediatric clinical settings nationally that choosing partial codes is an available option at additional pediatric care settings. The goal of this project was to inform institutional practices regarding the benefits, risks, and utility of partial code order options.

## 2. Materials and Methods

### 2.1. Patients

This three-year retrospective chart analysis was conducted at a large academic pediatric hospital that primarily cares for children with advanced cancer and other serious, life-threatening illnesses. This retrospective analysis included any patient with partial code order entry from 10 January 2022 to 10 January 2025. Data was extracted from the EPIC EHR by filtering clinical orders to identify all “Partial Code” orders placed for patients within the specified time range. The focus of this project was on cardiac and pulmonary arrest. In pediatric oncology, families may reasonably request non-invasive respiratory support only (e.g., positive pressure mask ventilation in the setting of progressive pleural effusions with a pulse). These non-arrest, non-code patient care scenarios were considered outside of the partial code project focus.

### 2.2. Methods

Patient demographics (gender, age, diagnosis, disease status, respiratory support) were obtained from the medical record. The electronic health record included three specific options for resuscitation orders during the three-year period covered by the chart review: 1. Do not attempt resuscitation (DNR); 2. Full resuscitation (the default order); and 3. Partial code. These three options were built into the medical record as the only three available resuscitation state orders. If an authorized clinician (physician or advanced practice provider) selected a partial code order, then a box automatically appeared in the electronic health record requiring the clinician to further clarify specific limitations on resuscitation. These limitations were categorized according to three categories: cardiac resuscitation, ventilation, and emergency drug protocol (Table 1).

Because these limitation categories were existent in a structured, consistent, accessible way in the medical record the project team used these exact categories a priori (convenience approach grounded in consistent data). The partial code order clarification box additionally required the clinician to mark the basis for the partial code order (as the patient’s preferences, the patient’s best interest, medical indication, and/or other) and to select presence or absence of a Physician Orders for Scope of Treatment (POST) form. POST forms are used institutionally as portable medical orders for seriously ill patients to detail end-of-life intervention preferences as actionable medical orders available for families across settings.

Partial code data were obtained through structured query language from the EHR. Categorization of the partial code order preferences was completed by downloading the specific order entries from the electronic health record for all patients with partial code orders. Descriptions of partial code preferences such as cardiac resuscitation, ventilation, drug protocol, and medical intervention; and concurrent POST were retrieved to capture clinician documentation. There was not a step of manual categorization or manual data extractions since this was an electronic health record downloaded by the informatics team.

This data was reported as categorical variables and counts. Two raters independently evaluated the partial code data set to look for consistencies and/or inconsistencies of partial code preferences using Excel sorting functions. The raters assessed which categories were selected for each patient and checked for internal consistency across all fields. They reached consensus through conversation. Evaluation of inter-rater reliability was performed using percentage agreement.

The Institutional Review Board deemed the “Code Orders, Documentation, and Experiences relevant to DNR” (CODED) Proposal (#25-2180) as Non- Human Subjects Research approved for Quality Improvement.

## 3. Results

A total of 206 code status modifications from default full code occurred with 93% DNR/DNI and 7% partial code orders entered. Fifteen partial code orders were entered on 12 patients (50% male and 50% female) over the three-year study period. Mean patient age at the time of partial code order entry was 14.4 years (median 17 years; range 9 months to 22 years). Demographics are provided in Table 2. Two independent data extractors obtained >95% inter-rater reliability across all data extraction items.

### 3.1. Documentation of Preferences

The inconsistencies of partial code preferences as captured in the EHR are provided in Table 3. While 93% of the electronic health records had a note within 24 h of the code order entry documenting the goals of care conversation, only half (53%) contained mention of discussion content covering all elements of the partial code resuscitation preferences. Institutional policy requires inpatient code status orders to additionally be uploaded as a scanned POST form at time of code status change. Most (67%) patients did not have a POST form in their chart. Two (17%) had a POST, but the documented preferences were listed as (“full treatment”) as compared to the limitations placed in the inpatient partial code order.

### 3.2. Basis for the Partial Code Order

Medical indications (53%), the patient’s best interest (40%), and the patient’s preferences (27%) were listed as the basis for the partial code order. Five patients had reached the age of majority and were legal decision-makers for their own code status. For two patients who had reached age of majority (18 and 19 years), patient preference was cited as the reason for the code status change. For two patients who had reached age of majority seemingly with capacity for life-sustaining treatment decisions at the time of the code status change (20 and 22 years), best interest and medical indications were listed as the reasons without reference to patient preference in the code order change. Patient preference was listed as the sole reason for code status change for two minor-aged patients (10 and 12 years).

### 3.3. Resuscitation Events

Only one patient with a partial code order underwent resuscitation. This resuscitation occurred two days after the partial code order was entered in the chart. In this resuscitation scenario, the child’s parent verbally rescinded any limits on resuscitation, and the child survived to return home. Ten (83%) patients with partial code orders are now deceased. Notably, four decedent patients (40%) had a change from partial code to full DNR <72 h after partial code entry. Death occurred an average of 14.6 days (median 13 days; range 0–40 days) after partial code order entry.

## 4. Discussion

Partial code orders were present in our free-standing children’s hospital, particularly among children in a terminal phase of illness with refractory or recurrent cancer. While these orders were entered with the intention of honoring patient and family preferences, in practice they may introduce complexity and confusion during resuscitation events [9]. In several cases, the specific combinations of code limitations appeared internally inconsistent. For example, some orders permitted the use of cardiac resuscitation medications but prohibited chest compressions, thereby voiding pharmaceutical benefits. Others allowed cardiac resuscitation but excluded intubation even though airway management and circulatory support are intrinsically linked in the absence of a pulse. Such inconsistencies violated the integrity of resuscitation algorithms and risked uncertainty among clinical teams, increasing the risk of hesitation and miscommunication in time-critical situations.

Partial codes appeared transient, as noted in the frequency of transition change to full DNR <3 days after initial partial code order entry for now-decedent patients. Ultimately, clinicians did not actually perform physiologically futile partial codes for any of the decedent patients. Confusion exists when goals of care are equated with code status. The partial code documentation did not universally reference patient preference as the basis for the partial code, most notably for the three patients at the age of majority who appeared to have decision-making capacity for life-sustaining decisions. There are realistic challenges to how clinicians may select this option in pediatrics (particularly for minors) where preferences are often articulated by parents or guardians and then framed in terms of best interest (another partial code selection category).

In practice, partial code orders seemed to function as a communication tool rather than a true resuscitation directive. Approximately half of the patients were already receiving positive pressure ventilation at the time the partial code was entered. For these patients, the cardiac resuscitation boxes were not checked and yet intubation/ventilation boxes were checked. This suggested the intent was to limit escalation to cardiac interventions rather than to alter existing respiratory support. The content of partial code orders for two of the already-ventilated patients contained free-text messages maintaining intubation but not escalating to cardiac interventions. The use of free text wording was not standardized, not readily visualized without additional button clicks, and risked delay or confusion in an urgent scenario. Ultimately, the code status should represent an evidence-based algorithm of resuscitation as a packaged entity.

In engaging with partners across the health care system regarding patient safety and resuscitation standard practices, the project team recognized that there may be a version of partial codes that make sense in the unique circumstance of resuscitation events during anesthesia. This is due to the potential impact of anesthetic medications and reversibility of arrest cause (such as, arrythmia recovery) with clinician experts readily at bedside. To honor this unique scenario and situation for which a partial code may make physiological sense and align with family goals, the project team worked together with anesthesiology colleagues to maintain a partial code option in the peri-procedural space (valid only during the peri-procedural timeframe). This limited occurrence of partial code included communication and documentation training with anesthesia and palliative care colleagues.

The clinical implications of removing partial code orders elsewhere in the health care system include improvements to resuscitation communication and code documentation consistency. Specifically, the removal of partial codes translates to a reduced risk of unclear guidance by reducing ambiguity (a real risk of medical errors) and fostering clarity. The primary clinical benefit is elimination of orders with negligible therapeutic benefit to the patient. Successful resuscitation requires rapid, coordinated effort across the code team in a full, ordered, evidence-based sequence of events. Code clarity is improved when the critical elements of a code are maintained and preserved across the health system. The clinical implication of removing partial code orders is the limitation of resuscitation options to evidence-based choices and therefore prevention of operational hazards.

Early and ongoing education for physicians and advanced practice providers as the authorized clinicians for order entry represents a necessary process consideration in preparing for and actionizing the removal of partial code orders. Bedside nurses benefit from inclusion in education as they are often the first on scene (or already present) for bedside events. Interdisciplinary colleagues (social workers, chaplains, child life specialists, etc.) benefit from education regarding the ethical, clinical, and communication considerations to foster shared insight and understanding across colleagues. Trainees benefit from learning about code integrity and code processes as part of their professional formation. Removal of partial code orders represents an opportunity to educate clinic staff as well as patients and families on the differences between pre-arrest scenarios and actual cardiopulmonary arrest. For example, a request to not intubate a patient with a chronic respiratory condition with a pulse and to instead support that patient with non-invasive positive pressure forms of ventilation is not a partial code. However, there is often baseline confusion and even a conflation between that concept of protection from endotracheal tube (in a non-arrest scenario) and intubation as part of a resuscitation algorithm in an arrest. Education and communication are key clarifiers. Implementation of the decision to remove partial code orders requires creating alternative frameworks to ensure that patient/family preferences in non-arrest scenarios remain clearly prioritized and implemented. Processes to ensure clarity, reliability, and accessibility of the order sets for non-arrest emergencies (example: clear order availability to document preferences for intubation for respiratory compromise with a pulse, preferences regarding non-invasive positive pressure ventilation, etc.) should occur prior to or concurrent with processes for removing partial code orders.

Clinicians should apply ethics-informed, process-based approaches centered on compassionate communication when families request interventions not anticipated to be physiologically beneficial for the child. Clinicians are not ethically obligated to provide treatments with no plausible benefit, even when requested by patients or families (example: “I want a code for my child but no chest compression or bagging”). These scenarios often involve familial misunderstanding of physiologic processes, resuscitation processes, and anticipated outcomes. The undercurrent behind such a request should be approached with humility and curiosity. Gently unpacking the intention behind the seemingly incongruent request offers a form of dignity and can transition toward shared decision-making informed not only by love but also by logic. The requester is likely asking the clinician to not give up on their child, to maintain hope in survival for the child, and to also not harm their child. Rather than lock in on the physiological facts, these requests for seemingly futile care extend opportunity to understand the values in tension. Interdisciplinary communication is key to explore potential areas for misunderstanding, attend to emotional reactions (shock, grief, distrust), and to contextualize toward shared values.

Partial code requests may represent a misunderstanding of resuscitation processes as families should not be expected to know the steps/components of a full resuscitation separate from informed conversations with clinical teams. Partial codes may also represent a therapeutic misestimation of resuscitation effectiveness (possibly based on common media depictions of CPR as heroic, calm, and promptly or even universally effective). Ultimately, requests for partial codes represent a chance to further explore values and preferences with patients/families. Clinicians may benefit from structured language guides which foster larger goals of care conversations when responding to partial code requests. A clinical implication of removing partial code orders is the practical opportunity to engage in patient-centric goals of care conversations and advance care planning. Specifically, patients and families benefit from prognostic clarity to inform goals of care which then contextualizes code conversations. A concern with partial codes is that they represent a piecemeal approach. The removal of partial codes presents a key opportunity to help transition a health system toward aligning resuscitation plans with patient values and realistic expectations about clinical outcomes rather than a piece-by-piece approach. A clinical implication of removing partial code orders is a necessary fostering of larger goals of care conversations. The larger hopes, values, and preferences of a patient cannot be captured in a code order. Instead, guiding clarity comes through advance care planning and clearly/succinctly communicating goals of care preferences with teams and in the electronic record.

A limitation of the project is its single institution location limits the generalizability of the findings. At other large pediatric hospitals, the prevalence of children with medical complexity and chronic critical illness may differ significantly, and this context may influence both the frequency and content of partial code orders. A strength is that these findings fostered pragmatic partnerships to collaborate in clinical improvement and patient safety action at the site. The project may serve as a model for baseline code status assessment for future translation of outputs. A limitation of this project was the lack of measured benefits of removing partial code orders beyond the advantages of reduced ambiguity in documentation. Transitions of health systems to binary code status do not necessarily translate to uniformly decreased invasive interventions without clear benefit to the patient. That is why code conversations must remain contextualized to both pre-arrest values and preferences and ultimately larger goals of care frameworks.

## 5. Conclusions

This project identified notable variability and inconsistency in the documentation and interpretation of partial code orders, highlighting potential risks to patient safety and communication clarity during critical events. The findings suggest that the current approach to partial code orders may not effectively support consistent, patient-centered, or goal-concordant care. Additional research is warranted to explore patient and family perspectives and workflow impacts (to include code status clarity at time of code events) following elimination of partial code orders.

The data collected through this project will inform institutional decisions regarding the development of safer, clearer standardized code status documentation practices. This includes transitioning away from the use of partial codes to enhance clarity and reduce ambiguity. This is in timely partnership with concerted educational efforts on communication/documentation surrounding larger goals of care prioritization. Engagement with patient safety, compliance, pediatric intensive care, oncology/hematology, nursing, anesthesia, palliative, and additional multidisciplinary colleagues is actively underway to gather perspectives and ensure that proposed policy and procedural changes are collaboratively developed, proactively communicated, and practically aligned with ethical and clinical best practices.

## Figures and Tables

**Table 1:** Partial Code Order Clarification Box (within the Electronic Medical Record).

Category	Clarification Checkbox Options
Cardiac Resuscitation	No chest compressions No defibrillation/cardioversion No internal/external pacemaker
Ventilation	No mechanical ventilation with intubation No bag/mask
Drug Protocol	No drug protocol after arrest occurs
Physician Orders for Scope of Treatment (POST) Form	Present Absent
Basis for the Order	Patient’s preferences Patient’s best interest Medical indication Other

**Table 2:** Demographics (n = 12).

Category	Response	n (%)
Birth Gender	Female	6 (50)
	Male	6 (50)
		
Age	Under 18 years	8 (66)
		
Underlying Diagnosis	Leukemia/Lymphoma	6 (50)
	Solid Tumor	4 (33)
	Brain Tumor	1 (8)
	Immunologic	1 (8)
		
Disease Status	Refractory	7 (58)
	Relapsed	5 (42)
		
Current Respiratory Support	Intubated on Ventilation	5 (42)
	Tracheostomy on Ventilator	1 (8)
	CPAP, BiPAP, nasal canula, or other	6 (50%)

**Table 3:** Partial Code Designations.

Partial Code Designation	Concern	n (%)
No chest compressions but yes to delivering cardiac medications	Illogical; medications will not reliably circulate without compressions in setting of cardiac arrest	7/12 (58%)
Yes chest compressions but no mechanical ventilation *	Decreases the efficacy and evidence-based of PALS algorithm	5/6 * (83%)
Yes chest compressions but no defibrillation/cardioversion	Defies PALS algorithm for initial management of tachycardia in unstable patient	1/12 (8%)
Yes internal/external pacing but no defibrillation/cardioversion	Inconsistent perception of electrical impulses to the heart	5/12 (42%)

* Note that six patients were already ventilated at time of partial code order entry and so the denominator is n = 6 to represent the patients not yet ventilated.

## Data Availability

The original contributions presented in this study are included in the article. Further inquiries can be directed to the corresponding author.

## Abbreviations

The following abbreviations are used in this manuscript:

DNR	do-not-resuscitate
CPR	Cardiopulmonary resuscitation
PALS	Pediatric Advance Life Support
POST	Physician Orders for Scope of Treatment
